# The neural basis of semantic cognition in Mandarin Chinese: A combined fMRI and TMS study

**DOI:** 10.1002/hbm.24781

**Published:** 2019-09-10

**Authors:** Qian Zhang, Hui Wang, Cimei Luo, Junjun Zhang, Zhenlan Jin, Ling Li

**Affiliations:** ^1^ Key Laboratory for NeuroInformation of Ministry of Education, High‐Field Magnetic Resonance Brain Imaging Key Laboratory of Sichuan Province, Center for Information in Medicine, School of Life Science and Technology University of Electronic Science and Technology of China Chengdu China; ^2^ School of Foreign Languages, Southwest Petroleum University Chengdu China

**Keywords:** fMRI and TMS, inferior frontal gyrus, posterior middle temporal gyrus, semantic cognition

## Abstract

While converging sources of evidence point to the possibility of a large‐scale distributed network for semantic cognition, a consensus regarding the underlying subregions and their specific function in this network has not been reached. In the current study, we combined functional magnetic resonance imaging (fMRI) and transcranial magnetic stimulation (TMS) methodology to investigate the neural basis of semantic cognition in Mandarin Chinese. In the fMRI experiment, strong activations were observed in left inferior frontal gyrus (IFG) and left middle temporal gyrus (MTG) for semantic judgment task. Moreover, functional connectivity was found from seed region left IFG to left MTG. Meanwhile, negative correlation between performance and extracted parameter estimates from left IFG to left MTG was detected in semantic task. Subsequent TMS stimulation over left IFG resulted in performance deficits in semantic judgment task, in contrast to other three sites: left MTG, right intraparietal sulcus (IPS) and a control site. We concluded that the neural basis of semantic processing for Mandarin Chinese closely resembled that for alphabetic languages such as English, supporting a language‐universal view on semantic cognition.

## 1 INTRODUCTION

Semantic cognition is fundamental to our mind and behavior: it refers to knowledge about people, words, objects, pictures, and faces and the use of this knowledge to drive behaviors that are appropriate for context and time (Corbett, Jefferies, Ehsan, & Ralph, 2009; Lambon Ralph & Patterson, 2008). Regarding the neural basis of semantic cognition, a large number of neuropsychological investigations (Corbett et al., 2009; Jefferies & Lambon Ralph, 2006; Noonan, Jefferies, Corbett, & Lambon Ralph, 2010), neuroimaging meta‐analyses (Noonan, Jefferies, Visser, & Lambon Ralph, 2013), and studies using inhibitory transcranial magnetic stimulation (TMS) (Davey et al., 2015; Whitney, Kirk, O'Sullivan, Lambon Ralph, & Jefferies, 2011, 2012) proposed a large‐scale distributed network including left inferior frontal gyrus (IFG), medial prefrontal cortex (mPFC), middle temporal gyrus (MTG), and lateral intraparietal sulcus (IPS) (Binder, Desai, Graves, & Conant, 2009; Jefferies, 2013; Noonan et al., 2013; Whitney et al., 2011). While large sources of evidence demonstrated clear roles performed by certain regions, the consensus regarding the underlying subregions that support semantic cognition and their specific function in this network has not been reached (Noonan et al., 2013). More importantly, these existing models of semantic processing were largely based on studies of Indo‐European languages (Hallam, Whitney, Hymers, Gouws, & Jefferies, 2016; Teige et al., 2018; Wagner et al., 2001).

Indeed, studies on Mandarin Chinese, an ideographic language, are inconsistent with those on alphabetic languages with regard to the neural basis of semantic cognition (Bolger, Perfetti, & Schneider, 2005; Tan, Laird, Li, & Fox, 2005; Wu, Ho, & Chen, 2012), in particular, it is not clear whether there is a language‐specific or a language‐general neural circuit of semantic processing across different languages. For example, in a meta‐analysis study, Tan et al. (2005) found that the left temporoparietal cortex has a unique contribution for the conversions from the grapheme to phoneme in alphabetic languages. Whereas, left middle frontal gyrus (MFG) plays a special role in Chinese language processing. In another meta‐analysis, a universal network across the writing systems was proposed by Bolger et al. (2005). What is more, some differential activations were also identified for Chinese processing, which was highly consistent with the meta‐analysis found in Tan et al.'s (2005).

However, using functional magnetic resonance imaging (fMRI) in a semantic task with words written in cursive font, Nakamura et al. (2012) demonstrated that two universal neural circuits, one for reading by eye and one for reading by hand, had similar activation and showed identical patterns of activation and repetition in Chinese and French language groups. Their findings suggested that languages with distinctive features of orthographies only regulated a fixed set of invariant macroscopic brain circuits, supporting a cross‐cultural commonality for semantic processing. More recently, another functional MRI study was conducted by Rueckl et al. (2015) to examine reading and speech perception in four highly different languages: Spanish, English, Hebrew, and Chinese. Using three complementary analytic approaches, they found small clusters of activation for Chinese in which speech‐print correlation was greater compared with other alphabetic languages. However, those clusters were not located in regions such as left middle frontal which had been claimed to be unique for Chinese language by former studies (Bolger et al., 2005; Tan et al., 2005). Taken together, there is still lack of controversy about the neural basis of semantic processing for Mandarin Chinese.

Most of recent studies on Chinese semantic processing have been performed by fMRI (Dong, Nakamura, Okada, Hanakawa, & Fukuyama, 2005; Rueckl et al., 2015; Tan et al., 2000; Yu, Mo, Li, & Mo, 2015; Zhang et al., 2017; Zhao et al., 2014), but these techniques may not be the best way to study systematically the specific role of each particular language area. Evidently, fMRI has been used to measure the whole‐brain activity to detect the cortical areas involved in certain brain undertakings. However, it cannot detect specific cortical area which plays a causal role in certain functions. Instead, by inducing focal and transient disruption of neural processing, the TMS technique is an effective tool to complement the results of fMRI (Pascual‐Leone, Tormos, & Keenan, 1998; Pascual‐Leone, Walsh, & Rothwell, 2000; Walsh & Rushworth, 1999). Thus, using a combined fMRI–TMS methodology, the present study aimed to investigate the neural basis of semantic cognition in Mandarin Chinese and the specific function of the implicated brain regions.

First, in the fMRI experiment, we sought to investigate the neural basis of semantic processing for Mandarin Chinese. In particular, we wondered whether there exists a similar large‐scale distributed network of semantic cognition to those of alphabetic languages or whether there exists some unique cortical regions merely for Chinese semantic processing. Then, to clarify the different functional roles of these regions, we used online TMS to generate focal “virtual lesions” in healthy volunteers to reveal whether these regions have causal relationships to the processing of semantic cognition in Chinese.

## 2 MATERIALS AND METHODS

### 2.1 Participants

Twenty healthy individuals participated in the fMRI study (10 males, mean age, 21.4 ± 1.96, range from 20 to 24 years) and another 6 subjects, along with the 20 participants in fMRI study, were selected for the subsequent TMS experiments. Two subjects quitted the TMS experiment later for personal reasons. Thus, altogether 24 subjects participated in the TMS study (16 males and 8 females, mean age, 21.63 ± 2.02, from 20 to 25 years). With normal or corrected‐to‐normal vision, all subjects neither had any personal or family history of neurological or psychiatric illness nor took medication in the course of the experiment. Before the formal experiment, they were all informed of the potential side effects of TMS and they offered written informed consent. All the subjects passed safety screening for TMS (Wassermann, 1998) and MRI. This study was approved by the local committee for the Protection of Human Subjects for the University of Electronic Science and Technology of China. The methods in our study were conducted in line with the approved guidelines and all experiments were in accordance with the declaration of Helsinki.

### 2.2 Experimental session design

**Figure 1:**
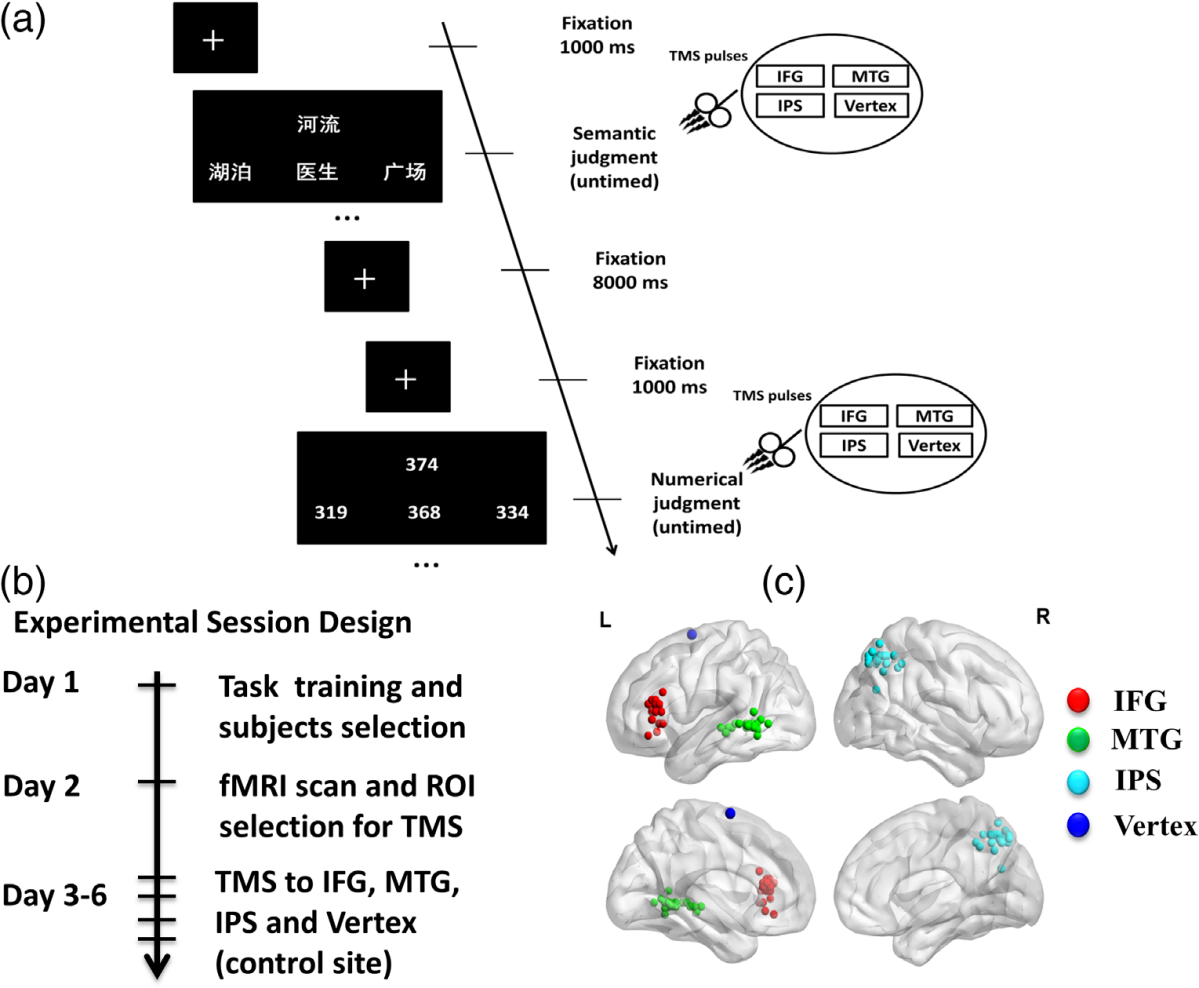
Experimental tasks, session design and TMS stimulation sites. (a) Experimental tasks and trial sequence. Each trial began with a fixation cross with 1,000 ms. Thereafter, subjects were required to choose either the target word that was semantically related with the cue word or the target number that was numerically close to the cue number. (b) Experimental session design. (c) The localization of IFG (red), MTG (green), and IPS (light blue) were slightly different for each subject on the basis of individual fMRI activations (each subject was represented by a dot). The approximate location of Vertex was depicted in dark blue. The average coordinates for each regions were: IFG: [−51, 23, 18]; MTG: [−57, −57, 0], IPS: [30, −54, 52], Vertex: [0, 0, 75], depicted on a standard template from MRIcro. fMRI, functional magnetic resonance imaging; IFG, inferior frontal gyrus; IPS, intraparietal sulcus; MTG, middle temporal gyrus; TMS, transcranial magnetic stimulation; ROI, region of interest [Color figure can be viewed at http://wileyonlinelibrary.com]

The experiments were carried out in 6 days (see Figure 1). On Day 1, participants received behavioral training for the semantic and numerical judgment tasks. Then, on Day 2 for the fMRI experiment, participants performed the same tasks and the functional neuroimaging results would be the basis for the location of the regions of interest (ROIs) to be stimulated in the subsequent TMS experiment. Finally, on the following Days 3–6, we applied online TMS at four cortical regions when participants performed the same tasks as those in the fMRI experiment. Three of the cortical regions were ROIs we got in the former fMRI experiment: left IFG, left MTG, and right IPS. The final region was Vertex as a control site, which was defined as position Cz according to the international 10–20‐system for EEG (Gold, Chang, Wang, Zhu, & Juan, 2014; Jung, Bungert, Bowtell, & Jackson, 2016; Sandrini, Umiltà, & Rusconi, 2011; Smittenaar, Fitzgerald, Romei, Wright, & Dolan, 2013) at Brodmann area 6. The order of TMS stimulation for the four cortical regions in Days 3–6 were counterbalanced across all participants and separated by at least a week.

### 2.3 Task

The presentation of task stimuli was performed by a PC with E‐Prime 2.0 software (Psychology software; Psychology Software Tools). All participants performed a semantic judgment task and a numerical judgment task (see Figure 1). The semantic judgment task was adapted from the previous fMRI and TMS studies (Binney, Embleton, Jefferies, Parker, & Lambon Ralph, 2010; Cao, Bin, Li, & Yan, 2014; Pobric, Jefferies, & Lambon Ralph, 2007, 2010a, 2010b, 2010c; Visser, Jefferies, Embleton, & Lambon Ralph, 2012). In this task, subjects were required to select a target word which is most related in meaning to a probe word from three words. Accordingly, each trial contained four written words: a probe word (e.g., 河流, river in English), the target word (e.g., 湖泊, lake in English), and two unrelated words (e.g., 医生, 广场, doctor and square in English). The four words used in each trial were matched for image ability (*p* = .42), word length (*p* = .65), and word frequency (*p* = .98). The numerical judgment task was also adapted from previous studies (Binney et al., 2010; Cao et al., 2014; Pobric et al., 2007; Pobric, Jefferies, & Lambon Ralph, 2010a, 2010b, 2010c; Visser et al., 2012). Similar to the semantic judgment task, in each trial, a probe number (e.g., 374) was shown on upper half of the screen and three choice numbers (e.g., 319, 368, and 334) were presented underneath. Subjects were asked to choose which of the three choice numbers was closest in value to the probe number.

### 2.4 fMRI procedure

Each participant performed two runs with mini‐block design in the fMRI experiment. Within each run, there were 15 semantic task blocks and 15 numerical task blocks. Those task blocks were ordered in random and interleaved by fixed blocks lasting for 8 s. Within each task block, a trial began with 1 s fixation followed by a word or a number stimuli presented for 3 s. Each task block consisted of four trials. Each run took 12 min. Subjects were required to select the target stimuli by pressing 1 of 3 designated buttons on a magnetic resonance compatible response box. Because of their small number of characters, Arabic numbers were presented in a larger font than semantic words (Arabic 1.6 × 1° visual angle; average of semantic words, 7.8× 0.8°).

#### 2.4.1 fMRI acquisition

MRI scanning was done using a 3.0 T GE Sigma scanner at the High‐Field Magnetic Resonance Brain Imaging Key Laboratory at the University of Electronic Science and Technology of China, Chengdu, China. We acquired functional MRI images using a gradient echo planar imaging (EPI) sequence. The scanning parameters were the following: 190 EPI volumes per block; TR = 2000 ms; TE = 30 ms; FA = 90°; FOV = 240 mm; matrix size = 64 × 64; voxel size = 3.75 × 3.75 × 3 mm^3^; 43 slices. We also acquired a high‐resolution, whole‐brain structural T1‐weighted image with a magnetization‐prepared gradient echo sequence. The scanning parameters were the following: TR = 1900 ms; TE = 2.26 ms; thickness, 1 mm; sagittal field of view, 256 × 256 mm^2^; flip angle, 9°; matrix, 256 × 256 × 176; voxel size, 1 × 1 × 1 mm^3^.

#### 2.4.2 fMRI data analysis

fMRI data analysis was performed using DPARSF version 4.1 (Data Processing Assistant for Resting‐State fMRI software; http://www.restfmri.net/forum/DPARSF) and SPM12 software (Statistical Parametric Mapping; Well‐come Trust Centre for Neuroimaging; http://www.fil.ion.ucl.ac.uk/spm). For each participant, the first five volumes of each functional time series were discarded for signal stabilization. Images were corrected for head movement between scans by an affine registration. The remaining functional images were preprocessed including slice timing correction, three‐dimensional motion correction, co‐registration to individual T1 structural image, and normalization by DARTEL (Ashburner, 2007) to the Montreal Neurological Institute (ICBM152 brain template) (http://www.bic.mni.mcgill.ca/ServicesAtlases/ICBM152NLin2009) reference space (3 × 3 × 3 mm^3^) and spatial smoothing with an 8 mm Gaussian kernel (full‐width at half maximum). Low‐frequency signal drifts were removed with a temporal high‐pass filter (default cutoff of 128 s). No subject had more than 2 mm of translation or 2° rotation.

At the single subject level, we operated statistical analysis on the basis of the context‐based general linear model (GLM) of the experiment. The individual trial onset sequence for semantic judgment task and numerical judgment task were obtained by convolving a canonical hemodynamic response function to form regressors of the design matrix (Gazzaley et al., 2007; Passaro et al., 2013; Robitaille et al., 2010; Yang, Fan, Wang, & Li, 2017).

At the group level, we first carried out paired *t* test to evaluate different activations between the semantic judgment task and the control judgment task (semantic‐number) and vice versa (number‐semantic) and between each task and the fixation blocks (semantic‐rest and control‐rest). Then, the following whole brain multi‐subject analysis was conducted by using a random effects model with a one‐sample *t* test on the summary statistic. The statistical contrast maps were thresholded at *p* < .001 (corrected for false discovery rate [FDR]) to control for multiple comparison (Benjamini & Hochberg, 1995; Genovese, Lazar, & Nichols, 2002).

#### 2.4.3 Defining ROIs for TMS targeting

We selected the ROIs according to contrast maps. All ROIs were identified on the peak activation of each cluster with 6‐mm radius sphere. Bold signal changes were analyzed according to the mean signal intensity of each ROI with the fixation epochs as a baseline.

Two ROI regions were localized at the left hemisphere of the frontal and temporal cortices, which were usually considered to be important in semantic judgment task. The average MNI coordinates across our subjects for left IFG was located at (*x* = −50.15 [*SD* = 4.96], *y* = 29.04 [*SD* = 4.03], *z* = 13.85 [*SD* = 4.93]) and for left MTG at (−57.43 [*SD* = 5.29], −47.29 [*SD* = 5.91], −1.68 [*SD* = 3.83]). Another region was located at parietal cortex in the right hemisphere, generally considered to be important in numerical judgment task across our subjects for right IPS (32.38 [*SD* =3.87], −65.22 [*SD* = 5.60], 47.71 [*SD* = 4.65]).

### 2.5 TMS stimulation

In the TMS experiment, online triple‐pulse TMS (tpTMS) was applied at the stimuli onset to transiently disrupt the processing of semantic cognition. According to the ROIs in the fMRI experiment, online tpTMS stimulation was administered with a Magstim super rapid magnetic stimulator and a figure‐of‐eight coil (diameter 70 mm) (Magstim Company Limited, Whiteland, United Kingdom). TMS session was conducted in accordance with the published safety guidelines (Rossi et al., 2009; Wassermann et al., 1996).

The coil was placed tangentially to the skull with the coil handle oriented perpendicular to the target cortex, guided by the online BrainSight frameless stereotaxy system (BrainSight Frameless, Rogue Research, Montreal, Canada). Triple‐pulse TMS was stimulated with at a pulse interval of 25 ms (40 Hz) at 100% resting motor threshold (RMT) as that used in our previous TMS studies on language comprehension (Zhang et al., 2018). tpTMS has proved to be more suitable for induction of measurable behavior effect size than single pulse TMS in higher cognitive functions and has thus been widely used in language cognition (Sack, 2005; Schuhmann, Schiller, Goebel, & Sack, 2009, 2012; Zhang et al., 2018). The RMT was identified immediately before the delivery of tpTMS. It was set as the stimulation intensity which had 50% chance to produce motor‐evoked potentials larger than 50 μV peak‐to‐peak in the contralateral first dorsal interosseous muscle, following stimulation over the hand area of the participant's right motor cortex (Wassermann et al., 1996; Yan, Wei, Zhang, Jin, & Li, 2016; Zhang et al., 2018). EMG activity was recorded with 9‐mm‐diameter Ag–AgCl surface cup electrodes and displayed on a conventional electromyography, which was also used to trigger the stimulator (Magstim Company Limited). Mean stimulation intensities for left IFG, left MTG, right IPS, and Vertex were 32.2 ± 5.0%, 32.3 ± 5.5%, 32.5 ± 5.5%, and 32.1 ± 5.0% of total stimulator output, respectively.

### 2.6 Statistical data analysis

Statistical data analysis was done using SPSS Statistics Release 20 (IBM, Somers, NY) GLM. Reaction time (RT) and accuracy rate (ACC) was measured. We used repeated measures ANOVA (Bonferroni corrected) to compare RT and ACC, with stimulation sites (IFG, MTG, IPS vs. Vertex) and tasks (semantic judgment task and numerical judgment task) as within‐subject factors. Post hoc *t* test, using Bonferroni corrected for multi comparisons, was conducted to compare the RT and ACC across different TMS sites within each task. The adjusted *p* value is .0083 for significant threshold.

### 2.7 Data availability

The authors confirm that all data underlying the findings are fully available without restriction. All relevant data are within the Supporting Information files.

## 3 RESULTS

We combined fMRI and TMS methodology to investigate the neural basis of semantic cognition in Mandarin Chinese. First, in the fMRI experiment, participants performed semantic and numerical judgment task in Mandarin Chinese. According to each subject's fMRI data, we identified three sites for TMS stimulation: left IFG, left MTG, right IPS, and a control site Vertex. Then, we delivered online triple‐pulse TMS (tpTMS) stimulation to each of these regions on separate days.

### 3.1 fMRI results

#### 3.1.1 Whole‐brain analysis

**Figure 2:**
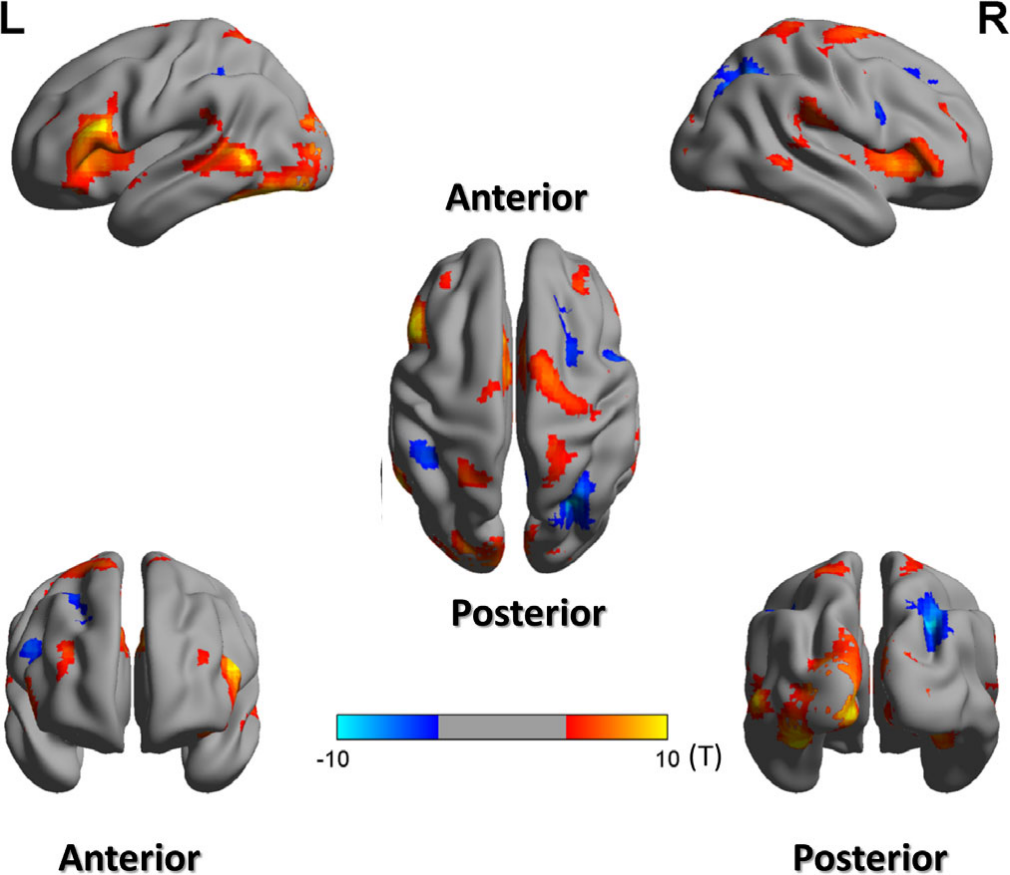
Brain activation maps for semantic and numerical judgment tasks. Activations displayed in the red/yellow color scale represent semantic > numerical judgment contrast, and blue color scale represent numerical > semantic contrast. These contrasts are survived at the cluster level, *p* < .001 (FDR‐corrected), with at least 100 voxels. FDR, false discovery rate [Color figure can be viewed at http://wileyonlinelibrary.com]

The data were initially processed as a whole brain cluster analysis, comparing semantic judgment tasks with numerical judgment tasks (semantics > numbers). The statistical image was evaluated at *p* < .001 (FDR‐corrected). The clusters that exceeded 30 voxels were shown in Table 1 and were also shown on the color scale in Figure 2. Activation was achieved in the ventrolateral frontal cortex of the left hemisphere, including the pars triangularis (BA45), the pars orbitalis (BA47), and also parts of the pars opercularis (BA44). We also obtained a cluster in the left MTG of the left hemisphere. In addition, a large cluster extending to the left and right occipital lobe (BA17/18) was observed, which may reflect the greater visual processing required by orthographic over digit stimuli or even semantic feedback to early visual areas (Hon et al., 2009).

**Table 1:** Activation regions for the whole brain general linear model (GLM) analysis

			MNI coordinates			
Brain region	Hemisphere	Cluster	*x*	*y*	*z*	*t* score
Semantic > numerical task						
Inferior frontal gyrus	L	506	−51	24	18	10.63
			−33	30	6	8.19
			−42	15	18	8.39
Inferior frontal gyrus	R	148	27	33	6	8.96
Superior frontal gyrus	R	59	15	3	72	6.43
			18	−6	66	6.31
Middle frontal gyrus	R	31	33	45	27	5.35
			33	54	27	5.30
Middle temporal gyrus	L	199	−57	−57	0	9.38
			−57	−36	0	5.70
			−42	−45	9	6.47
Middle temporal gyrus	R	36	66	−42	0	5.49
Insula	R	134	33	27	−3	8.14
			39	18	3	7.68
Middle Cingulum	L	136	−6	−3	30	8.66
Anterior Cingulum	L	48	−6	15	30	7.87
Middle Cingulum	R	122	9	9	33	7.06
			15	−33	42	5.78
Superior parietal gyrus	L	30	−18	−60	69	6.99
Numerical > semantic task						
Inferior parietal gyrus	R	385	30	−66	39	9.23
			33	−60	48	6.76
Precentral gyrus	R	39	51	9	30	7.58
Inferior parietal gyrus	L	104	−45	−42	45	5.50
			−54	−33	42	4.87
Middle frontal gyrus	R	116	27	6	51	5.95
Superior frontal gyrus	R	50	24	24	45	4.53
Precuneus	R	64	9	−57	18	5.71
Cerebelum	L	30	−24	−69	−33	4.75

Contrasting the numerical task against the semantic task (numerical > ), we found activation in the bilateral inferior parietal lobe, the right precuneus, the right frontal cortex (middle/superior gyrus), and the right superior occipital gyrus (corrected to *p* < .001 using FDR).

**Figure 3:**
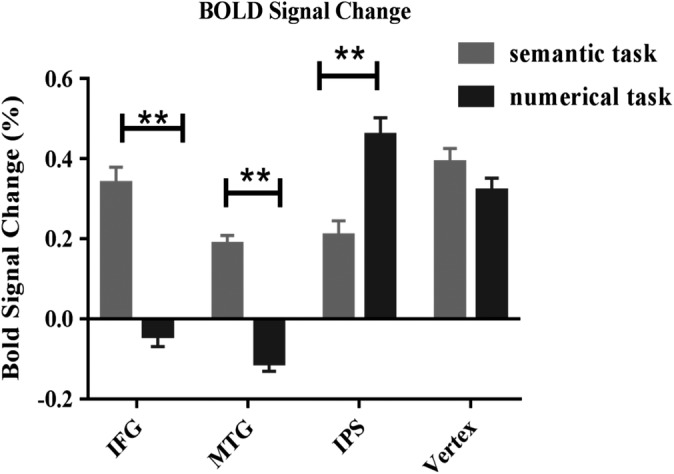
The BOLD signal change in two experimental conditions extracted from 6 mm regions of interest (ROIs) for left IFG, left MTG, right IPS, and Vertex. The percentage signal change for semantic judgment and numerical judgment task were represented by gray and black bars, respectively. Error bars correspond to the *SE*, **p < .001 Bonferroni corrected. IFG, inferior frontal gyrus; IPS, intraparietal sulcus; MTG, middle temporal gyrus

The two‐way repeated ANOVA (Bonferroni corrected) (4 ROI × 2 task) ROI analysis showed a significant main effect of ROI (IFG, MTG, IPS, and Vertex) (*F* [3, 57] = 27.528, *p* < .001), a main effect of task (semantic vs. numerical task) (*F* [1, 19] = 62.258, *p* < .001) and a significant interaction between ROI and task (*F* [3, 57] = 137.074, *p* < .0001) (See Figure 3). To identify the source of this two‐way interaction, we used paired *t* test, Bonferroni corrected for multiple comparisons, to compare the effects of the four ROIs (IFG, MTG, IPS, and Vertex) under the semantic and numerical task conditions. The results revealed that left IFG was more active during the semantic judgment task (*t* [19] = 13.038, *p* < .0001), than during numerical judgment task. Similar to left IFG, strong activation in left MTG was found in semantic judgment task, compared with that in numerical judgment task (*t* [19] = 10.556, *p* < .0001). In contrast, right IPS was more active during numerical task (*t* [19] = −9.059, *p* < .0001). Finally, although Vertex showed strong BOLD signal change in semantic and numerical judgment task, respectively, no difference of BOLD signal change was detected between the two tasks (*t* [19] = 2.562, Bonferroni corrected *p* > .05).

#### 3.1.2 Functional connectivity analysis

To explore whether any observed core regions worked in concert with other regions as a network for cognitive processing, we used generalized psychophysiological interactions (PPI) approach (gPPI; https://www.nitrc.org/projects/gppi; McLaren, Ries, Xu, & Johnson, 2012) for the functional connectivity analysis.

According to the whole brain analysis, four ROIs (IFG, MTG, IPS, and Vertex) were taken as seed regions with a 6 mm radius sphere centered at the peak activation coordinates. To test the network of cognitive processing, two PPI contrasts were set: (semantic task > numerical task) and (numerical task > semantic task). First, for the semantic > numerical contrast, increased connectivity were only found between seed region left IFG and left MTG (MNI coordinates: −57, −42, 0, *p*_(uncorrected)_ = .001, cluster size = 66, *t* = 3.58). No other significant connectivity was detected with seed regions (MTG, IPS, and Vertex), respectively. For the numerical > semantic contrast, there was no significant functional connectivity detected for the four seed regions.

**Figure 4:**
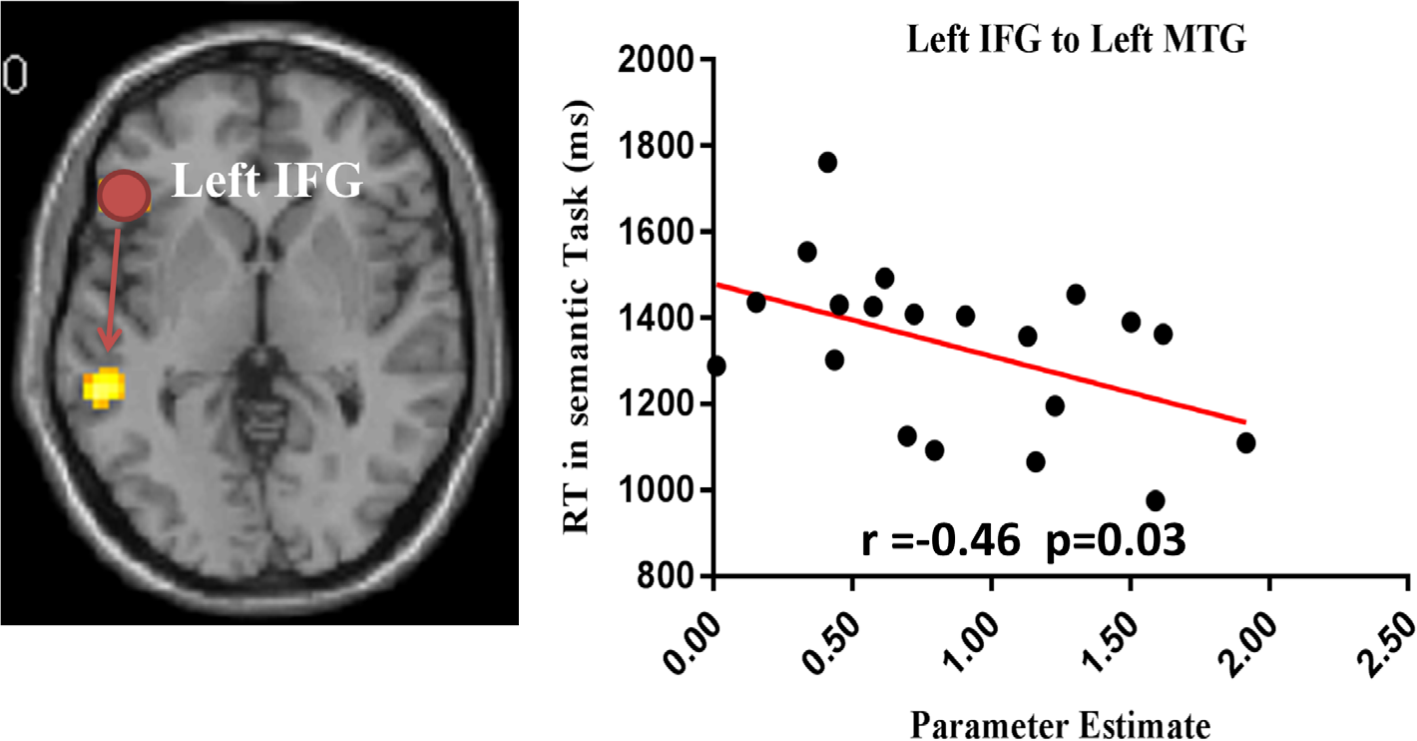
The correlation analysis between extracted parameter estimates from left MTG using left IFG as seed region and reaction times in semantic judgment task. The red line indicates that the correlation was significant (*p* < .05, corrected). IFG, inferior frontal gyrus; MTG, middle temporal gyrus [Color figure can be viewed at http://wileyonlinelibrary.com]

Additionally, we further performed correlation analysis between participants' behavior performance and extracted parameter estimates of the regions with functional connectivity. Negative correlation between RTs and extracted parameter estimates from left IFG to left MTG was detected (*r* = −.46, *p* = .03) in semantic task (Figure 4). These results indicate that the stronger the functional connectivity between left IFG and left MTG, the faster it is for the subjects to finish the semantic task and the better their performance. No other correlations were found between performance and parameter estimates in semantic or numerical judgment task.

### 3.2 TMS results

**Figure 5:**
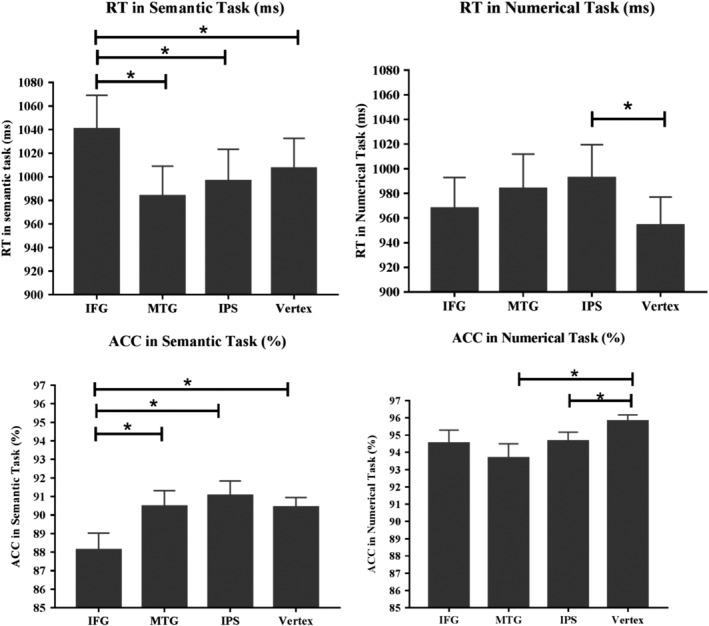
TMS effects. Reaction time (RT) and accuracy rate (ACC) after TMS stimulation to left IFG, left MTG, right IPS, and Vertex in semantic and numerical judgment task, respectively. The asterisk means Bonferroni corrected *p* < .05. Error bars denote *SE*. IFG, inferior frontal gyrus; IPS, intraparietal sulcus; MTG, middle temporal gyrus

The TMS‐induced effects in RT and ACC were shown in Figure 5 for semantic and numerical judgment task, respectively.

#### 3.2.1 TMS‐induced changes in RT

Subjects' mean RT was examined with two‐way repeated measures ANOVA (Bonferroni corrected). The ANOVA model included the factors: task (semantic vs. numerical) and TMS site (IFG, MTG, IPS, and Vertex). Mean RT and ACC for the semantic and numerical judgment task at four TMS stimulation sites are listed in Table 2.

**Table 2:** The average RT (ms) and ACC (%) for four TMS stimulation sites

TMS stimulation sites	IFG	MTG	IPS	Vertex
RT (ms), mean (*SEM*)				
Semantic task	1,040.46 (24.67)	983.59 (25.51)	996.40 (25.53)	1,007.22 (19.40)
Numerical task	967.85 (25.08)	983.73 (24.18)	992.43 (24.16)	954.16 (22.90)
ACC (%), mean (*SEM*)				
Semantic task	88.11 (0. 92)	90.47 (0. 85)	91.04 (0. 80)	90.41 (0. 53)
Numerical task	94.53 (0. 76)	93.67 (0. 82)	94.65 (0. 52)	95.80 (0. 38)

Abbreviations: ACC, accuracy rate; IFG, inferior frontal gyrus; IPS, intraparietal sulcus; MTG, middle temporal gyrus; RT, reaction time; TMS, transcranial magnetic stimulation.

Overall, the two‐factorial ANOVA revealed a main effect of task [*F* (1, 23) = 7.567, *p* < .05], indicating that RTs were longer when subjects made semantic judgment task compared to those in numerical judgment task. We also found an interaction between task and TMS site [*F* (3, 69) = 15.273, *p* < .001]. Accordingly, post hoc *t* test, using Bonferroni corrected for multiple comparisons, was conducted to compare the RTs difference after TMS stimulation over four TMS sites within each task. For the semantic judgment task, the results showed that RTs were significantly prolonged after TMS stimulation over IFG, compared with those over MTG [*t* (23) = 3.406, *p* = .002], IPS [*t* (23) = 2.734, *p* = .012], and the control site, Vertex [*t* (23) = 2.927, *p* = .008]. For the numerical task, significantly increased RTs were found after TMS stimulation over right IPS, compared to similar stimulation over Vertex [*t* (23) = 2.591, *p* = .016]. No significant differences in RTs were found between IFG, MTG, and Vertex in numerical judgment task (*p* > .05 for all pairwise comparisons).

#### 3.2.2 TMS‐induced changes in ACC

A two‐factorial ANOVA with task (semantic task vs. numerical task) and TMS sites (IFG, MTG, IPS, and Vertex) as the two within‐subject factors revealed a significant main effect of task [*F* (1, 23) = 108.611, *p* < .05], indicating that ACC was significantly higher when subjects made numerical judgments compared to semantic judgments. Exploratory analyses also demonstrate a significant main effect of TMS sites [*F* (3, 69) = 3.413, *p* < .05] and interaction between task and TMS sites [*F* (3, 69) = 11.598, *p* < .001]. For the semantic task, post hoc *t* tests revealed decreased ACC at IFG site compared with other three sites, MTG [*t* (23) = −3.286, *p* = .003], IPS [*t* (23) = −4.831, *p* = .001], and Vertex [*t* (23) = −2.578, *p* = .017]. No significant differences in ACC were found between MTG, IPS, and Vertex (*p* > .05 for all pairwise comparisons). For the numerical task, post hoc *t* tests revealed decreased ACC at IPS site compared with Vertex [*t* (23) = −3.093, *p* = .005]. Interestingly, we also found decreased ACC at MTG site compared with Vertex for numerical task [*t* (23) = −3.254, *p* = .003].

## 4 DISCUSSION

The present study aimed to investigate the neural basis of semantic cognition in Mandarin Chinese and the specific functions of its underlying subregions using a combined fMRI–TMS methodology. First, in the fMRI experiment, strong activation in left IFG and left MTG were detected in semantic judgment task, coupled with significant functional connectivity between these regions. Meanwhile, functional connectivity analysis between left IFG and MTG showed strong correlation with performance in the same task. Interestingly, no significant activation was found in left MFG and superior parietal gyri, which were claimed to be unique for Chinese language processing. Moreover, TMS stimulation over left IFG largely disrupted performance in semantic task, compared to the other three sites: left MTG, right IPS, and Vertex. However, no such deficits were found at left MTG in the same task.

### 4.1 The role of left IFG in Chinese semantic cognition

An extensive set of studies on alphabetic languages such as English have shown that left IFG plays an essential role in regulating semantic activation (Badre & D'Esposito, 2009; Badre & Wagner, 2007; Demb et al., 1995; Gabrieli, Poldrack, & Desmond, 1998; Gold & Buckner, 2002; Thompson‐Schill, 2003). For example, left IFG activation largely increases when participants identify words with weak associations or accessing words with ambiguous meaning (Badre, Poldrack, Paré‐Blagoev, Insler, & Wagner, 2005; Noppeney, Phillips, & Price, 2004; Wagner, Pare‐Blagoev, Clark, & Poldrack, 2001). Moreover, patients with damage on left IFG showed greater semantic deficits on similar tasks which require high executive control demands. These findings suggest that the prefrontal cortex is mainly associated with semantic control process (Corbett et al., 2009; Corbett, Jefferies, & Lambon Ralph, 2011; Jefferies, Patterson, & Ralph, 2008; Noonan et al., 2010; Novick, Kan, Trueswell, & Thompson‐Schill, 2009; Soni et al., 2009; Teige et al., 2018). More recently, studies applying TMS on healthy participants were conducted to investigate the causal role of left IFG in semantic cognition (Whitney et al., 2011). In a recent TMS study, using material in English, Whitney et al. (2011) investigated the specific roles of left IFG and MTG in semantic processing in two different manipulations of semantic cognition (associative strength and feature selection). Their data analysis revealed that left IFG along with MTG support both the controlled semantic retrieval underpinned by a bottom‐up automatic spreading activation mechanism and the feature selection of semantic knowledge which involved a more top‐down executive mechanism.

With similar semantic retrieval task, the work presented here confirmed not only the recruitment but also the causal role of left IFG for the regulation of semantic processing for Mandarin Chinese. In the fMRI experiment, we found that left IFG was one of the crucial regions that were significantly activated in semantic judgment task, compared with that in numerical task. Moreover, the neural activity of left IFG was substantially greater in semantic task compared to that in numerical task as reflected in BOLD signal change of this region. Meanwhile, functional connectivity between left IFG and other regions such as left MTG revealed the multi‐recruitment of those regions in the network of semantic cognition. In the online TMS study, significant increases in RT were observed after TMS stimulation over left IFG in semantic task, in contrast to the other sites: MTG, IPS, and control site Vertex. Consistently, stimulation at left IFG also significantly decreased ACC for semantic judgment task, as opposed to that in numerical judgment task. Taken together, the current study is in agreement with the studies on alphabetic languages that the left IFG plays a causal role in accessing, retrieving, and executively manipulating semantic knowledge for Mandarin Chinese.

### 4.2 The role of left MTG in Chinese semantic cognition

There is great uncertainty about the function of posterior temporal cortex in semantic cognition. Early evidences from functional imaging studies on alphabetic languages mainly focus on the role of left MTG as a store for semantic knowledge (Binder et al., 2009; Gold & Buckner, 2002; Hickok & Poeppel, 2004; Indefrey & Levelt, 2004). However, neuropsychological studies on semantic aphasia (SA) patients failed to find converging evidence with previous fMRI work. For instance, irrespective of whether they have lesion on MTG plus other temporal and inferior parietal cortex, SA patients are able to access semantic knowledge, indicating that this region does not exclusively act as a key semantic store (Davey et al., 2016; Jefferies & Lambon Ralph, 2006; Whitney et al., 2012). Recent evidence from semantic dementia (Corbett et al., 2009; Noonan et al., 2010), as well as functional neuroimaging studies of healthy individuals (Davey et al., 2016; Jefferies & Lambon Ralph, 2006; Schwartz et al., 2009) have also noted that instead of a passive store for semantic attributes, left MTG may be involved in the strategic retrieval of semantic information.

First, in line with the above fMRI studies on semantic control at left MTG, we identified greater activation for Chinese semantic judgment task at this region along with inferior regions of the left frontal context. In addition, PPI analysis revealed strong functional connectivity between left IFG and MTG, coupling with correlation with performance in semantic task, further supporting the view that left MTG works in concert with left prefrontal cortex in order to allow strategic access of semantic information (Friederici, 2009). Therefore, the present fMRI findings in Mandarin Chinese are consistent with the recruitment of MTG, alongside IFG, for semantic processing (Badre et al., 2005; Davey et al., 2015, 2016; Noonan et al., 2013; Poldrack et al., 1999; Snijders et al., 2009).

However, in the online TMS results study, TMS‐induced changes for semantic selection task at left MTG were not observed as expected. One possible interpretation for the discrepancy between fMRI and TMS results may lie in the different contribution of those sites in semantic control network. Davey et al., (2016) investigated the different functional roles of IFG, MTG, and IPS in semantic cognition with a comparison with the meta‐analysis of Noonan et al. (2013). Their data provided converging evidence for the three components of semantic cognition: (a) default mode network (DMN) preferentially for the automatic spreading activation with semantic representation, which is supported by ATL and other regions (Jackson, Hoffman, Pobric, & Lambon Ralph, 2016; Lau, Gramfort, Hämäläinen, & Kuperberg, 2013; Power & Petersen, 2013; Wirth et al., 2011); (b) multiple‐demand executive network (MDN) (Duncan, 2010) for top‐down allocation of attention and usually supported by frontoparietal control systems (Power & Petersen, 2013); (c) a third network including left IFG and left MTG for required goal‐driven retrieval. Particularly, their findings show that left MTG is located at the intersection of the DMN and MDN, a position that would allow it to integrate information from two anticorrelated large‐scale systems implicated in automatic semantic processing and executive control. Although SA patients with left IFG and left MTG lesions have highly similar deficits, cases with left IFG lesions have greater difficulty inhibiting previously relevant semantic information, leading to more difficulty in semantic selection and retrieval (Gardner et al., 2012; Jefferies, Baker, Doran, & Ralph, 2007). Likewise, it is reasonable that TMS‐induced effects over left MTG were not observed due to the less contribution of MTG (compared with that of left IFG) in the process of semantic retrieval. Thus, our analysis is consistent with the studies on alphabetic languages that left MTG, coupling with left IFG, are essential components of a distributed cortical network underpinning executive semantic processing.

## 5 CONCLUSION

Our study investigated the neural basis of semantic cognition in Mandarin Chinese and the specific functions of its underlying subregions using a combined fMRI–TMS methodology. We have demonstrated that left IFG specifically plays a causal role in semantic cognition, along with subordinate recruitment of other regions such as MTG. These findings are convergent with others studies on alphabetic languages. Therefore, we propose that the neural basis of semantic processing for Mandarin Chinese closely resembled that for alphabetic languages such as English, supporting a language‐universal view on semantic cognition.

6

## CONFLICT OF INTERESTS

The authors declare no conflict of interest.

## Supporting information

Supporting Information files

**S1** Brain activations displayed in the red color scale represent semantic > numerical judgment contrast. These contrasts are survived at the cluster level, *p* < .001 (FDR‐corrected), with at least 100 voxels. FDR, false discovery rate.

*S2* Brain activations displayed in the red color scale represent numerical > semantic judgment contrast. These contrasts are survived at the cluster level, *p* < .001 (FDR‐corrected), with at least 100 voxels. FDR, false discovery rate.

## Data Availability

The authors confirm that all data underlying the findings are fully available without restriction. All relevant data are within the Supporting Information files.
