# Supplementary figures and images for: The neural basis of semantic cognition in Mandarin Chinese: A combined fMRI and TMS study
Source: Hum Brain Mapp. 2019 Sep 10;40(18):5412–23. doi: 10.1002/hbm.24781 (PMC6864898; doi:10.1002/hbm.24781)

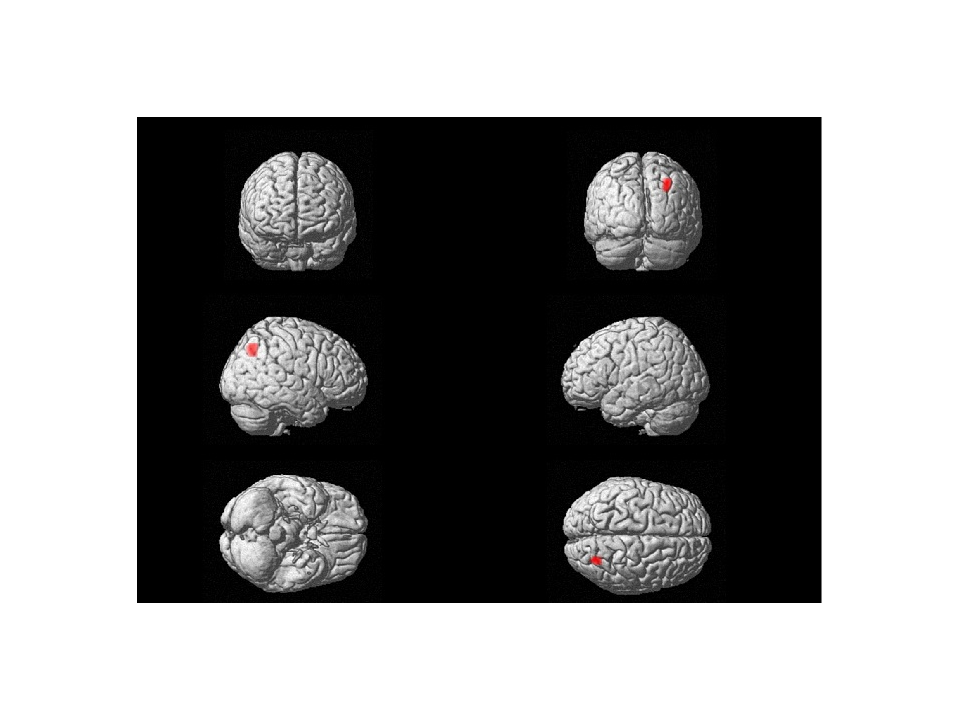

Supplement: Supplementary file 2 — S1 Brain activations displayed in the red color scale represent semantic > numerical judgment contrast. These contrasts are survived at the cluster level, p < .001 (FDR‐corrected), with at least 100 voxels. FDR, false discovery rate. [file HBM-40-5412-s002.tif]

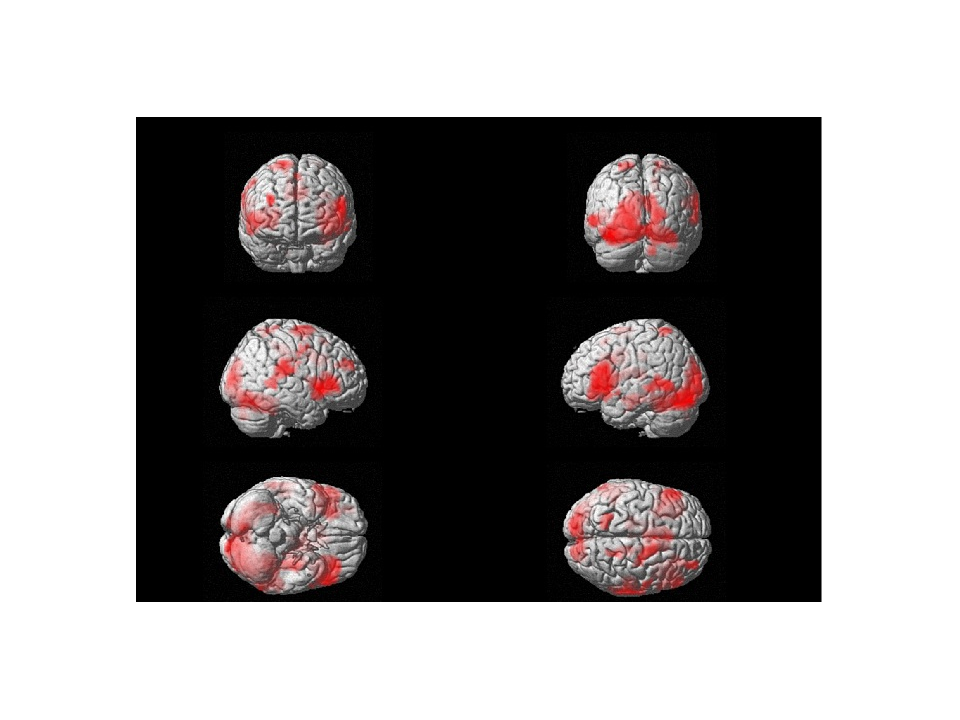

Supplement: Supplementary file 3 — S2 Brain activations displayed in the red color scale represent numerical > semantic judgment contrast. These contrasts are survived at the cluster level, p < .001 (FDR‐corrected), with at least 100 voxels. FDR, false discovery rate. [file HBM-40-5412-s003.tif]
